# Thermoelectric materials developments: past, present, and future

**DOI:** 10.1080/14686996.2021.1966242

**Published:** 2021-12-20

**Authors:** Takao Mori, Antoine Maignan

**Affiliations:** a International Center for Materials Nanoarchitectonics (WPI-MANA), National Institute for Materials Science, Tsukuba, Japan; b Graduate School of Pure and Applied Sciences, University of Tsukuba, Tsukuba, Japan; c Laboratoire de Cristallographie et Sciences des Matériaux (CRISMAT), Normandie Université, Caen, France

**Keywords:** Thermoelectricity, energy conversion, materials for energy, thermal conductivity, energy harvesting

It is almost exactly 200 years since Thomas Seebeck discovered the Seebeck effect. Thermoelectric materials can convert thermal energy to electricity via the Seebeck effect, and they are attractive because this conversion can be achieved with a compact solid state semiconductor device. The field of thermoelectrics is now at an exciting time in history, with new high-performance materials and novel enhancement principles being discovered [1–3], innovative module development being carried out, and the long-held dream of widespread power generation applications appearing to be closer to realization than it has ever been before. There are two increasingly important applicative directions in which thermoelectric materials can play a large and critical role. One is the urgent necessity for energy saving [4] to contribute to the recent carbon neutral/zero emission goals for the environment, and the other is energy harvesting to dynamically power the myriad necessary sensors for the Internet of Things (IoT) future society [5].

This Focus Issue in *Science and Technology of Advanced Materials (STAM)* focuses on ‘Thermoelectric Materials’ and has gathered a number of notable papers from 2019 to 2021. Two of the STAM Young Scientist Award Winners (the Thermoelectric Symposium E-MRS2019, Nice, France, May 2019) contributed two papers to this issue. Thermoelectric generators are based on pairs of n- and p-type legs. In the paper by Wenjie Xie et al., the scandium substitution in NbCoSn has been shown to be efficient to generate a high power factor for a p-type thermoelectric half Heusler, which is the weak leg of thermoelectric pairs made on such intermetallic materials [6]. The prototypical commercial thermoelectric material is Bi_2_Te_3_, a long time discovered material but still difficult to overbeat in terms of thermoelectric performance. This is why research on its properties improvement is still a very actual topic. In particular, reducing the size of its particles is a way to degrade its thermal conductivity and thus increase its thermoelectric figure of merit. In that respect, the well-crystallized and oriented nanowires obtained by electrodeposition, reported in the paper by Cristina V. Manzano et al., demonstrate the importance of this growth process for the synthesis of Bi_2_Te_3_ nanowires with both good aspect ratio and crystallographic orientation responsible for the variation of the mean free path of the acoustic phonons, and hence the low thermal conductivity as compared to bulk crystals of the same material [7].

The NIMS Award 2020 was on the general theme of thermal energy and was dually awarded to two researchers, Profs. Hiroshi Julian Goldsmid and Kunihito Koumoto, both of whom achieved seminal breakthrough developments on thermoelectric materials. Prof. Goldsmid has been considered as the Father of Thermoelectrics owing to his discovery and development of the excellent thermoelectric properties of bismuth telluride in 1954. Bismuth-telluride-type materials have been the champion performing thermoelectric materials at room temperature for almost 70 years, and they are presently still the only widely applied materials as Peltier coolers. He also discovered the bipolar effect in thermal conductivity in 1958 and observed the phonon drag effect in 1959. Following these seminal discoveries, he has continually contributed to the development of the thermoelectrics field for many decades. For this Focus Issue, Prof. Goldsmid contributed a review paper from a unique perspective on the strategies for improving the thermoelectric figure of merit, *ZT* = *S*^2^*T*/*ρκ*, where *S* is Seebeck coefficient, *ρ* is electrical resistivity, *κ* is thermal conductivity and *T* is absolute temperature [8]. The larger the value that *ZT* takes, the closer the thermoelectric conversion efficiency approaches the ideal Carnot efficiency.

Prof. Koumoto has made striking advancements in the development of environmental-friendly and earth-abundant thermoelectric materials. The classical high-performance thermoelectric materials such as bismuth telluride, lead telluride, etc. tend to contain very rare or toxic elements. Prof. Koumoto led the early development in achieving high thermoelectric performance in oxides and sulfides, through novel principles such as nanoblock integration, artificial superlattice structures, etc. His notable work on hybridization via organic molecules intercalation in layered sulfides achieved flexible high-performance thermoelectrics, which were targeted for wearable IoT power generation. For this Focus Issue, Prof. Koumoto and coworkers contributed an original paper on the recent development of a novel abundant sulfide thermoelectric material [9].

Finally, the guest editors, Profs. Maignan and Mori, and coauthors contributed a paper focused on how spin entropy can enhance thermoelectric performance [10]. The utilization of magnetism to enhance the Seebeck effect and the thermoelectric power factor is a recent especially hot topic in the thermoelectric field. It has been found that in addition to the spin entropy [11], magnetic interactions [12] and spin fluctuations [13] also demonstrate enhancement effects on thermoelectric performance. As such, new magnetic semiconductor thermoelectric materials have been discovered [14] the increasing motivation for developing high-performance and viable thermoelectric materials for applications in both substantial energy savings and energy harvesting sensors, we hope that this STAM Focus Issue on Thermoelectric Materials can inspire further striking developments in the field.
